# First-Principles Study on Si Atom Diffusion Behavior in Ni-Based Superalloys

**DOI:** 10.3390/ma16175989

**Published:** 2023-08-31

**Authors:** Yubo Sun, Zhiping Wang, Mingrun Du, Yimeng Du, Wang Zhang

**Affiliations:** 1 School of Materials Science and Engineering, Shenyang University of Technology, Shenyang 110870, China; yb-sun@cauc.edu.cn; 2 School of Aeronautical Engineering, Civil Aviation University of China, Tianjin 300300, China; mrdu@cauc.edu.cn (M.D.); 17858322875@163.com (W.Z.)

**Keywords:** Si atom diffusion behavior, energy barrier, Ni-based superalloy, melting point depressant elements, refractory elements, first-principles

## Abstract

The Si atom diffusion behavior in Ni-based superalloys was evaluated based on first-principles calculations. Also, the site occupation of Si atoms as the melting point depressant elements in Cr, Mo, and W atom doped γ-Ni and γ′-Ni_3_Fe supercells was discussed and Si atom diffusion behaviors between both adjacent octahedral interstices were analyzed. Calculation results indicated that formation enthalpy (∆*H_f_*) was decreased, stability was improved by doping alloying elements Cr, Mo, and W in γ-Ni and γ′-Ni_3_Fe supercells, Si atoms were more inclined to occupy octahedral interstices and the diffusion energy barrier was increased by increasing the radius of the doped alloy element. Especially, two diffusion paths were available for Si atoms in the γ′-Ni_3_Fe and Si diffusion energy barrier around the shared Fe atoms between adjacent octahedral interstices and was significantly lower than that around the shared Ni atoms. The increase of interaction strength between the doped M atom/octahedron constituent atom and Si atom increased Si atom diffusion and decreased the diffusion energy barrier. The Si atom diffusion behavior provides a theoretical basis for the phase structure evolution in wide-gap brazed joints.

## 1. Introduction

An aeroengine turbine guide vane made of Ni-based superalloys is prone to crack under high-temperature, high-pressure, and thermal shock alternating the load environment. Wide–gap brazing (WGB) is usually applied to repair such structures based on isothermal solidification induced by the melting point depressant (MPD) element diffusion. The quantity, morphology, and distribution of porosity defects and eutectic structure with hard brittle characteristics in brazed joints have significant effects on the mechanical properties of these joints [1,2,3]. Eutectic structures are formed due to insufficient diffusion of MPD elements in liquid–phase brazing material during the cooling process [4,5,6,7,8]. For instance, MPD elements B and Si can easily synthetize M_3_B, MB boride, and Ni_3_Si silicide, which have high hardness and poor toughness and can easily cause stress concentration resulting in the reduction of high-temperature mechanical properties in brazed joints of Ni-based superalloys [9,10].

WGB joints are mainly consisted of γ + γ′ phases and some alloy elements (such as Cr, Mo, and W) and are added to the filler materials to enhance joint structure uniformity and high-temperature mechanical properties. Also, the occupation of alloy elements was found to affect the diffusion of MPD elements [11]. Ji et al. [12] studied the influences of refractory elements M (Mo, W, Re, and Ta) on the diffusion characteristics of B in Ni-based single-crystal superalloys and showed that B atoms preferentially occupied γ-Ni and γ′-Ni_3_Al octahedral interstices. Research findings indicated that the above refractory elements doped in structures, prevented the diffusion of B and B diffusion E _(Barrier from product)_ strength change trend, were in the following order: Re < Mo < W < Ta. Evaluation of electronic structures showed that various atomic radii of Re to Ta and different hybrid orbitals among M *d* and B *2p* from Re to Ta played key roles in B diffusion properties in Ni-based single-crystal superalloys. This research clarifies refractory metal effects on B diffusion behavior, as the MPD element in γ-Ni and γ′-Ni_3_Al provides theoretical basis for designing filler materials for WGB joints in Ni-based superalloys. Chen et al. [13] studied the γ/γ′/γ″ system interface properties, and alloying element strengthening mechanisms, on the γ/γ″ and γ′/γ″ coexisting interfaces by first-principles calculations and showed that the most thermodynamically stable interface structure was the Ni (center) + X (termination) matching configuration. Multi-interface doping findings demonstrated that Tc, Ta, Mo, Re, and W preferably substituted Nb-cp positions and Os, Co, and Fe tended to occupy the Ni-fc position. Re and W had the strongest influence on the improvement of γ/γ″ and the γ′/γ″ interface bonding strength synergistically. Their findings could help develop new Ni-based superalloys simultaneously strengthened by the γ′ + γ″ phases. Gong et al. [14] applied first-principles to investigate the strengthening influences of W and Re alloying elements on Ni_3_Al and concluded that improved chemical bonding among W/Re alloying atoms and neighboring Ni host atoms was the key strengthening mechanism in W/Re-alloyed Ni_3_Al systems. They also reported that the thermodynamic and mechanical characteristics of the alloying element W in Ni_3_Al were similar to those of Re, which suggested the possibility of replacement of Re with W in Ni-based single-crystal superalloys. Elements, such as Re and W, had strengthening effects on the phase structure of Ni-based high-temperature alloys; therefore, these elements are also commonly used in Ni-based alloys. However, their effects on the diffusion of MPD elements in wide-gap brazing joints were not yet clear. Chen et al. [15] studied the influences of adding γ (FCC)-forming (Cr and Si, for oxidation resistance) and γ′ (L12)-forming (Ta, Ti, and Ni, for creep property) elements on the creep properties, oxidation resistance, and microstructural stability of two-phased γ/γ′ Co-based superalloys based on Co-Al-W systems. Liu et al. [16] found that the oxidation resistance of Fe-Si alloy was increased by increasing Si content below 1150 °C, while increasing Si content of the alloy resulted in higher oxidation rates above 1150 °C due to the formation of liquid fayalite. Therefore, Si enhanced the high-temperature performance of Ni-based superalloys and is a common MPD element in wide-gap brazed joints. However, the effects of refractory metals on Si diffusion have been rarely reported in wide-gap brazed joints of Ni-based superalloys. Bridges et al. [17] proposed a modified Hart’s equation for predicting the effective interdiffusion coefficients of nanomaterials that accounts for surface diffusion in addition to lattice and grain boundary diffusion. Their finds indicated that the diffusion coefficients calculated by the modified Hart’s equation agree with the values determined by Sauer–Friese analysis for nanoparticles. Zeng et al. [18] used Electron Probe MicroAnalysis to determine the interdiffusion coefficients of fcc Ni-Cu-Mo alloy at temperatures of 1273, 1373, and 1473 K based on the concentration distribution of solid–solid diffusion couples and re-evaluated the kinetic parameters of fcc Ni-Cu-Mo alloy. The atomic mobility of fcc Ni-Cu-Mo alloys were optimized as a function of the temperature and composition by means of CALculation of Thermo-Physical Properties program. The calculated diffusion rate, concentration distribution, and diffusion path strongly agree with the experimental results for the further understanding of the variation of kinetic phenomena, which can lead to a better understanding of the changes in dynamic phenomena. Chen et al. [19] fabricated 12 groups of single-phase (gamma and gamma′) Ni-Al-Cr diffusion couples to measure the concentration profiles at 1423, 1473, and 1523 K using the electron probe microanalysis technique. Atomic mobilities of the γ phase in the Ni-Al-Cr system were established by the means of High-throughput Determination of Interdiffusion Coefficients software, which was confirmed by the coincidence between the calculated and experimental properties, including the composition profiles and diffusion paths based on the atomic mobilities together with the available thermodynamic descriptions; hence, the three-dimensional interdiffusivities planes at 1423–1523 K were constructed. Mustafa Azeem et al. [20] investigated the effect of the grain boundary on the diffusion of a phosphorous atom in alpha-Fe by means of molecular dynamics simulations. Their results provided an insight into the grain boundary trapping effect in alpha-Fe. To sum up, it provides theoretical guidance for further revealing the diffusion behavior of atoms in nickel-based alloys.

In this study, we investigated the effect of elements (Cr, Mo, and W) doped in γ-Ni, γ’-Ni_3_Fe on the formation enthalpy using first-principles calculations, and we also discussed the site preferences of Si atom doped in the supercell of γ-Ni, γ′-Ni_3_Fe_,_ and analyzed the energy barrier of Si atom between both isovalent sites. The aim was to reveal the diffusion behavior of Si atom in γ-Ni, γ′-Ni_3_Fe and obtain the diffusion coefficient of Si atom under high-temperature for future experimental investigations, which might be beneficial for the design of braze filler materials.

## 2. Computational Details

In this research, systematic DFT calculations were performed with Vienna Ab initio Simulation Package (VASP) [21,22] and climbing image nudged elastic band (CI-NEB) [23] approaches to investigate the effects of alloy elements (M = Cr, Mo, and W) on the diffusion of Si as MPD elements in γ-Ni and γ′-Ni_3_Fe. Interactions among ions and core electrons were defined based on the projector augmented wave (PAW) method [24,25] and Perdew–Burke–Ernzerhof parameterization of generalized gradient approximation [26] was applied for the exchange correlation potentials. Monkhorst and Pack k-point meshes were applied to perform Brillouin zone integrations [27]. After effective test calculations, cutoff energy was set at 400 eV for γ-Ni and γ′-Ni_3_Fe phases. Ionic and electronic step convergence conditions in self-consistent calculations were set at 0.01 eV/Å and 10^−5^ eV, respectively. CI-NEB calculations were performed for the determination of diffusion barriers and transition state, with maximum force break condition on each atom lower than 0.003 eV/Å. 4 × 4 × 4 k-point meshes created according to Monkhorste–Pack scheme was applied for numerical integrations over γ-Ni and γ′-Ni_3_Fe Brillouin zones. Furthermore, since Ni-based single-crystal (Ni-SCS) superalloys had a magnetic nature, spin–restriction was adopted for the calculation from Ref. [12]. In addition, the contour maps of partial density of states (PDOS) were obtained and evaluated [28].

**Figure 1:**
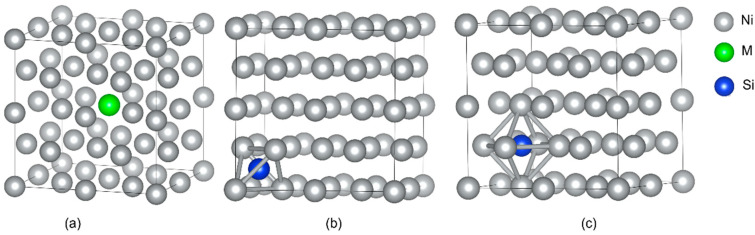
The supercell of γ-Ni with the occupancy of alloy element M and Si doping. (**a**) The model of alloy element M doped in γ-Ni, (**b**,**c**) represent Si doping in tetrahedral and octahedral interstice, respectively.

**Figure 2:**
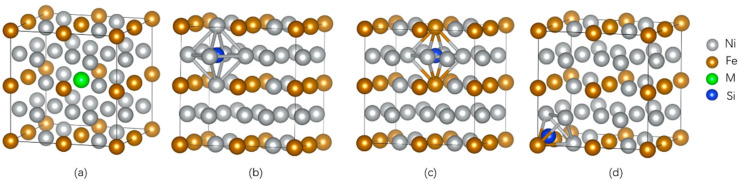
The supercell of γ′-Ni_3_Fe with the occupancy of alloy element M and Si doping. (**a**) The model of alloy element M doped in γ′-Ni_3_Fe, (**b**,**c**) represent Si doping in two kinds of octahedral interstice, respectively, (**d**) represent Si doping in tetrahedral interstice.

The supercells of dimensions 2 × 2 × 2 were established for γ-Ni and γ′-Ni_3_Fe with space groups of *Fm-3m* and *Pm-3m*, respectively, where the central Ni/Fe atom was substituted by the M atom (Cr, Mo, and W) [12]. The Si as MPD element was doped in tetrahedral and octahedral interstices. In addition, there were two types of octahedral interstices in γ′-Ni_3_Fe supercells, including ones composed of 6 Ni atoms, 4 Ni atoms and 2 Fe atoms, as shown in Figure 1 and Figure 2.

The formation enthalpy (∆*H_f_*) of substituted M elements and the formation energy (∆*E*) of Si as MPD element doping in the supercell were calculated by Equation (1) [29,30] and Equation (2) [31,32]:

(1) $$∆H_{f}=[E(\mathrm{Ni}(32-x-y)\mathrm{FexMy})-(32-x-y)E(\mathrm{Ni})-xE(\mathrm{Fe})-yE(M)]/32$$

(2) $$∆E_{f}=[E(\mathrm{Ni}(32-x-y-1)\mathrm{FexMySi})-(32-x-y-1)E(\mathrm{Ni})-xE(\mathrm{Fe})-yE(M)-E(\mathrm{Si})]$$

where *E*(Ni(32 − x − y) and *E*(Ni(32 − x − y − 1) (x = 8 or 7, y = 1) are total energies of γ-Ni or γ′-Ni_3_Fe with M centered and Si doped in the supercell, respectively. Also, *E*(M), *E*(Ni), and *E*(Si) are per atom energies.

## 3. Results and Discussion

### 3.1. Structural Characteristics

The structure was optimized to obtain the lattice parameters of γ-Ni and γ′-Ni_3_Fe in the stable ground state and the findings are summarized in Table 1. After optimization, γ-Ni and γ′-Ni_3_Fe were both face-centered cubic with α = β = γ = 90° and the calculation results of γ-Ni32 strongly agreed with theoretical results [12,33,34]. ∆*H_f_* of γ-Ni with centered Ni atom substituted by M was negative and was further decreased by increasing the radius of M. This indicated that the supercells were stable and could be synthesized easily.

Few studies are available on the first-principles of γ′-Ni_3_Fe, γ′-Ni_3_Fe. Therefore, calculated results obtained in this work were compared to those of γ′-Ni_3_Al, which are taken from the literature. The radius of Al (1.43 Å) is bigger than that of Fe (1.27 Å); thereby, the lattice parameters of γ′-Ni_3_Al were higher than those of γ′-Ni_3_Fe. In this research, γ′-Ni_3_Fe calculated results were smaller than those of γ′-Ni_3_Al, which were reliable. The ∆*H_f_* of γ′-Ni_3_Fe with M (Cr, Mo, and W) doped were gradually decreased and that of γ′-Ni_3_Fe with W was negative. This indicated that the thermodynamic stability of doped supercells was further enhanced.

### 3.2. Occupying Tendency of Si Atoms as MPD Elements in γ-Ni and γ′-Ni_3_Fe

The ∆*E* of Si atom doping in tetrahedral and interstices were 1.7291 and 0.8325 eV, respectively, as shown in Table 2. The lattice parameters and the volume of supercells with Si atom doping in tetrahedral interstices were higher than those in octahedral interstices. This indicated the occurrence of significant lattice distortion and Si atom occupying the tetrahedral interstitial site needed to overcome large lattice distortion forces. Hence, Si atoms tended to occupy the octahedral interstitial sites of γ-Ni making the resulting structure more stable.

The ∆*E* values of Si atom doping in tetrahedral and octahedral interstices were 2.7038, 2.1744 (O1), and 1.6601 (O2) eV, respectively, and ∆*E* difference between O1 and O2 was minimal. The two octahedral interstices were applied as the initial and final positions for Si atom diffusion. Also, the lattice constants of Si atom-doped supercells in O1, O2, and T positions were all 7.0747 Å, while those of Si occupying octahedral and tetrahedral interstices in γ-Ni were 7.0615 and 7.1576 Å, respectively. It was clear that lattice distortion due to Si atom doping in γ′-Ni_3_Fe supercell was lower than that due to doping in γ-Ni, which further indicated that Si atoms more stably occupied the octahedral interstitial sites of γ′-Ni_3_Fe supercells.

### 3.3. Si Atom Diffusion Behaviors in γ-Ni and γ′-Ni_3_Fe Supercells

#### 3.3.1. Si Atom Diffusion Behavior in γ-Ni Supercells

**Figure 3:**
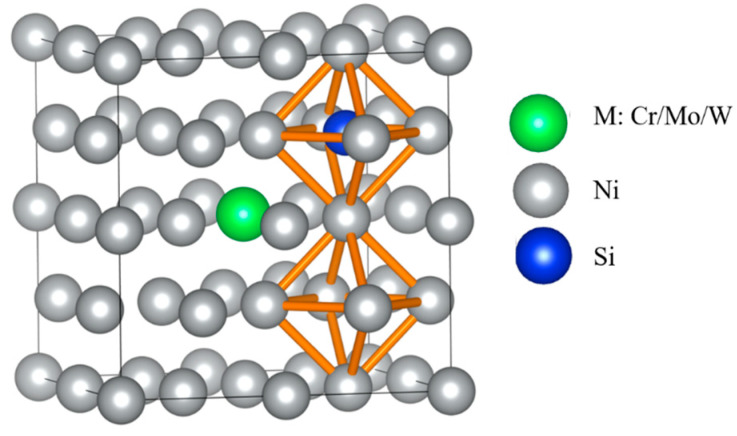
Schematic diagram of Si atom located at the initial and final positions of octahedral interstitials in γ-Ni supercells with alloy elements M.

According to Table 2, two adjacent octahedral interstitials closest to M atom were taken as initial and final positions of Si diffusion, which were isovalent sites, as presented in Figure 3. Four diffuser sub-position points were inserted between initial and final positions for the calculation of the Si atom diffusion energy barrier and characterized possible diffusion pathways.

**Figure 4:**
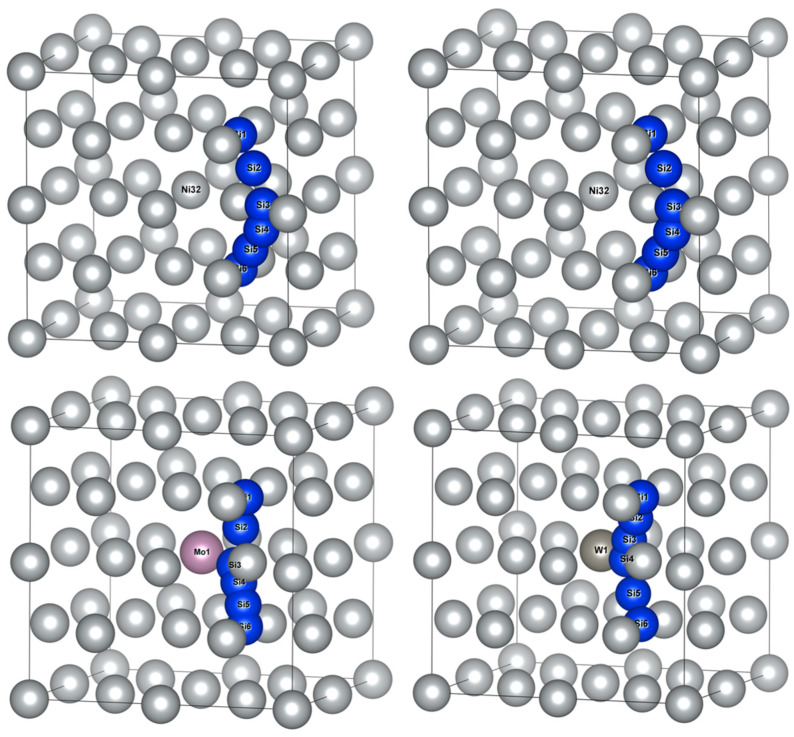
Schematic diagram of Si atom diffusion pathways between the initial and final positions of octahedral interstitials in γ-Ni supercells with alloy elements M.

The schematic diagram of Si atom diffusion pathways in Ni32, Ni31Cr, Ni31Mo and Ni31W supercells are illustrated in Figure 4. Si1 and Si6 represented the initial and final positions of diffusion pathways, respectively. Si2~Si5 represented transition state positions of diffusion pathways, whose coordinate positions were obtained from the calculated results. It should be noted that Si atoms affected the positions of adjacent Ni atoms during diffusion, i.e., the surrounding Ni atoms were in dynamic change. Therefore, Figure 4 applied Ni atom coordinates of γ-Ni-doped alloy element M, combined with Si atom coordinates of diffusion calculations, to facilitate the observation of Si atom diffusion paths.

By comparing Si atom diffusion paths, Si atom diffusion pathways in Ni32 and Ni31Cr showed outward diffusion away from the central Ni/M atom, while the diffusion pathways of Si in Ni31Mo and Ni31W presented inward diffusion near the central M atom. The identification of diffusion pathway as away from, or biased, the central atom of supercell depends on the distance between the central atom and Ni atom near the inflection point along the diffusion path. Measurement results were 2.5135, 2.5213, 3.5235, and 3.5270 Å for Si atom diffusion pathways in Ni32, Ni31Cr, Ni31Mo, and Ni31W, respectively. Therefore, diffusion pathways appeared biasing toward the central atom in Ni31Mo and Ni31W due to the large gap formed surrounding the central atom when Mo and W were doped. 

**Figure 5:**
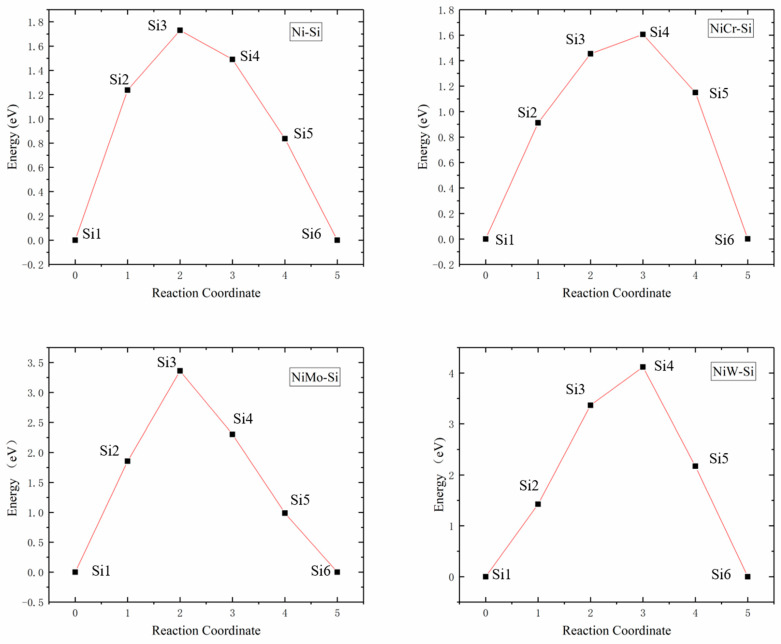
Energy barrier versus reaction coordinate of Si atom diffusion in Ni32, Ni31Cr, Ni31Mo, and Ni31W supercells.

The Si atom diffusion energy barriers in Ni32, Ni31Cr, Ni31Mo, and Ni31W supercells were 1.7299, 1.6061, 3.3601, and 4.1194 eV, respectively, as presented in Figure 5. Since the radii of Ni and Cr atoms were similar, their energy barriers and diffusion pathways were also similar, while the radii of Mo and W atoms were larger than those of Ni and Cr atoms, resulting in higher lattice distortions and diffusion energy barriers. Increase of the radius of the central atom of supercells increased lattice internal stress, which in turn increased Si atom diffusion energy barriers, was not favorable to Si atom diffusion.

#### 3.3.2. Si Atom Diffusion Behavior in the γ′-Ni_3_Fe Supercell

**Figure 6:**
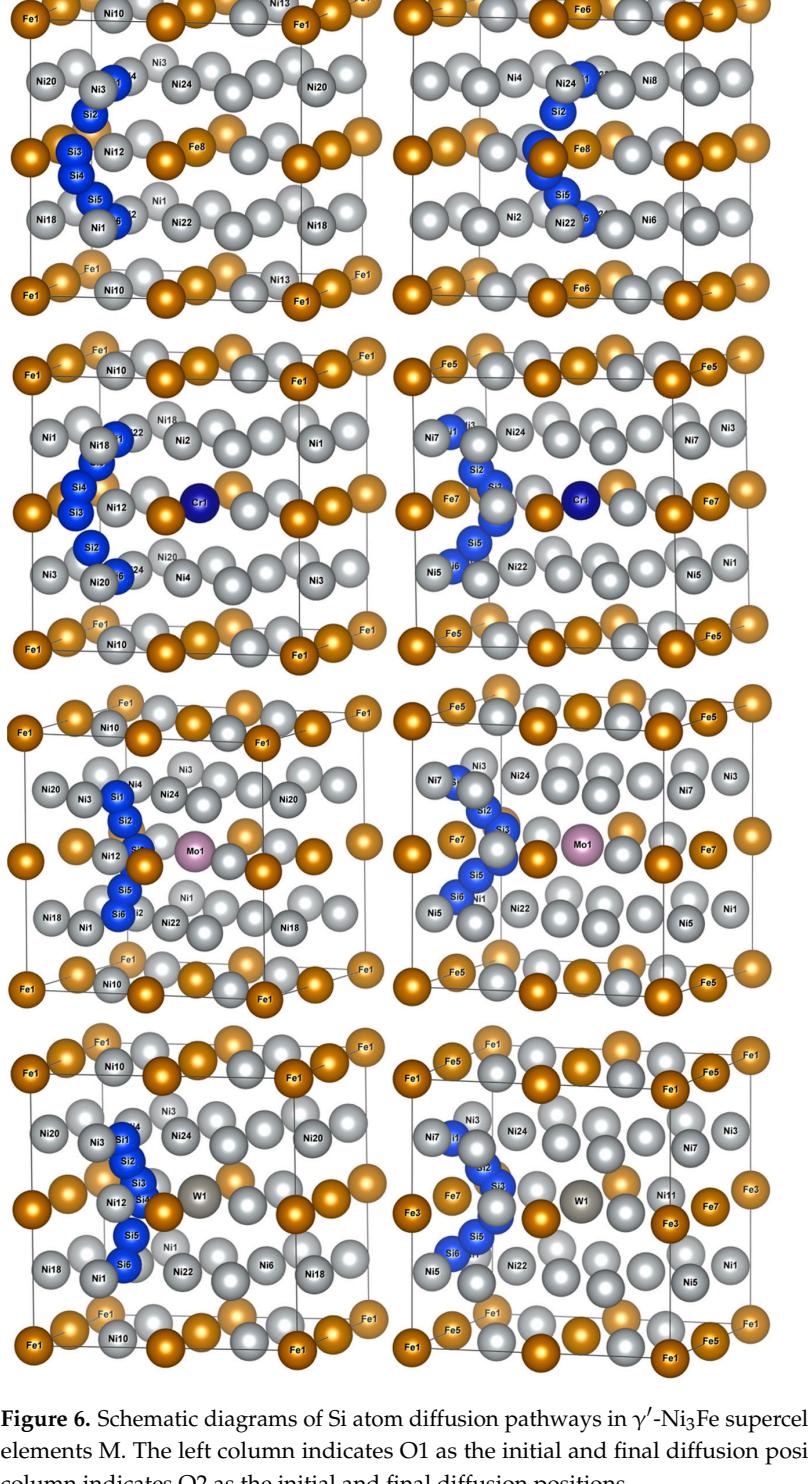
Schematic diagrams of Si atom diffusion pathways in γ′-Ni_3_Fe supercells doped with alloy elements M. The left column indicates O1 as the initial and final diffusion positions and the right column indicates O2 as the initial and final diffusion positions.

The calculations of diffusion energy barriers were performed based on O1 and O2 as the initial and final positions of Si atom diffusion, respectively, as presented in Figure 6. The left column had O1 as the initial and final positions of diffusion, i.e., an octahedral interstice composed of six Ni atoms, and the right column had O2 as the initial and final positions of diffusion, i.e., an octahedral interstice composed of two Fe atoms and four Ni atoms.

The two Si atom diffusion paths revealed that the Si atom diffused around the shared Ni12/Fe8(7) atom of two isovalent octahedral interstices, as illustrated in Figure 6. When O1 was considered as the initial and final position of the diffusion, Si atom diffusion paths were basically the same as those in γ-Ni, and Si diffusion paths presented a diffusion behavior away from Fe8 and Cr1 when no-doped and doped with Cr, respectively. When Mo and W were doped, Si atoms presented a diffusion path toward Mo1 and W1, respectively. When O2 was considered as the initial and final diffusion position, Si atom diffusion path also presented a diffusion behavior toward central Cr atoms when doped with Cr, which was different from that in γ-Ni.

**Figure 7:**
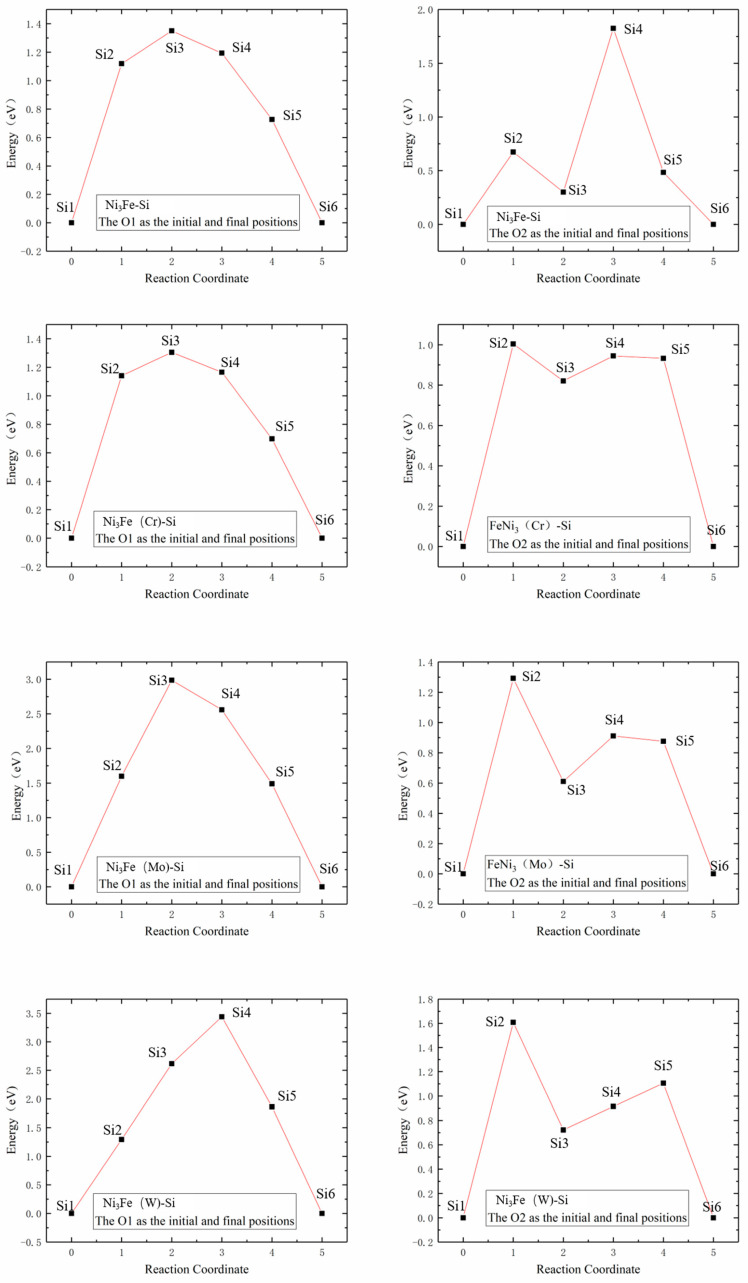
Energy barrier versus reaction coordinate of Si atom diffusion in γ′-Ni_3_Fe supercells doped with alloy elements M. The left column indicates O1 as the initial and final position of diffusion and right column indicates O2 as the initial and final position of diffusion.

Figure 7 illustrates Si atom diffusion energy barriers in γ′-Ni_3_Fe supercells, where the left column shows Si atom diffusion energy barriers at the O1 interstitial site, and the right column shows that at the O2 interstitial site. The comparison of energy barrier diagrams revealed that: (1) the energy barrier diagrams in the left column showed single-peak energy barrier distributions, while the right column revealed approximate double-peak energy barrier distributions; and (2) the Si atom energy barriers in undoped, Cr, Mo and W doped γ′-Ni_3_Fe supercells were 1.3499, 1.3048, 2.9863, and 3.4415 eV for O1 as the initial and final diffusion position, respectively. Energy barriers and energy barrier distribution were similar for undoped and Cr doped structures, since the radii of Cr and Ni atoms were almost similar, while their energy barriers were significantly higher when doping with Mo and W atoms with the increase of the radius of the doped atom. Si atom energy barriers of undoped and Cr, Mo, and W doped γ′-Ni_3_Fe supercells for O2 as the initial and final diffusion position were 1.8242, 1.0035, 1.2924, and 1.6084 eV, respectively, where doping with alloying elements significantly reduced Si atom energy barrier for diffusion and energy barrier value after doping the alloying elements was also increased with the increase of the radius of alloying elements. It is worth noting that diffusion energy barriers regarding O2 as the initial and final diffusion position were significantly lower than those when O1 was used as initial and final diffusion position. This further indicated that Si atoms tended to occupy O2 octahedral interstices which were composed of Fe and Ni atoms. Furthermore, the formation enthalpy and total energy with Si doping at O2 position were also at their lowest when Si was doped in γ′-Ni_3_Fe, which was consistent with the calculation results presented in Table 2.

#### 3.3.3. Relation between Si Diffusion Properties and Electronic Structure

**Figure 8:**
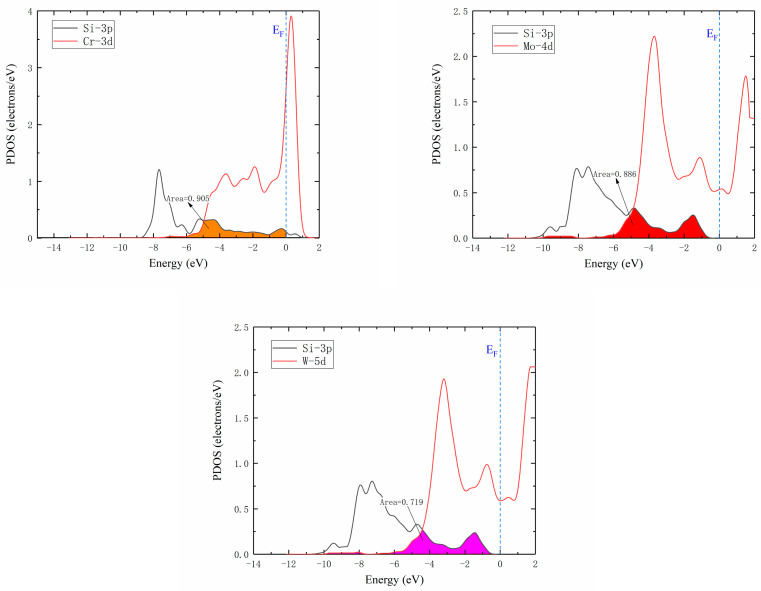
Partial density of states. Colored areas show overlapped hybrid orbitals between M (Cr, Mo, or W) *d* and Si *3p*. *E_f_* is defined as the value of the total density of states at the energy of 0.

The Si atom diffusion energy barrier was affected by electron interactions in different orbitals and charge transfer with Si atom located at the saddle point. The partial density of states (PDOS) of saddle point for the diffusion of Si atoms between the two adjacent octahedrons in γ-Ni supercells doped with M are illustrated in Figure 8. As known, the interaction strength between M-Si was determined by the electron number, which was decreased with the loss of electrons [12]. The strength of interaction between M and Si atoms was characterized by the overlapping of doping M *d* Si *3p* hybrid orbitals. It was seen that interaction strength between Cr and Si was the strongest, followed by Mo-Si, and then by W-Si ranks which was the weakest according to overlapping areas, which revealed that Si atoms around interstices lost fewer electrons from Cr to W. This indicated that the effect of each of the doped Cr, Mo, and W atoms on Si atom diffusion was gradually weakened, which strongly agreed with previously calculated results for the diffusion barrier of Si diffusing in M-doped γ-Ni supercells. Therefore, the increase of interaction strength decreased diffusion energy barrier, which was more conducive to diffusion.

**Figure 9:**
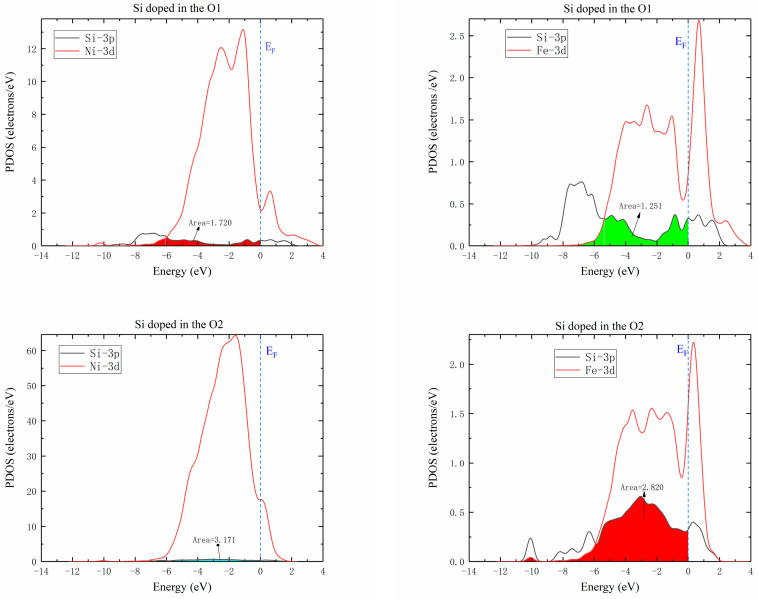
Partial density of states of Ni/Fe *d* Si *3p* hybrid orbitals when Si atom was doped in O1 and O2 octahedral interstices of γ′-Ni_3_Fe.

Figure 9 shows the partial densities of states of Ni-Si and Fe-Si when Si atoms were doped in O1 and O2 octahedral interstices of the γ′-Ni_3_Fe supercell. The comparison of the overlap areas of Ni/Fe *d* and Si *3p* orbital hybridization revealed that interaction between Ni/Fe and Si atoms at the O2 position was significantly higher than that at the O1 position. Therefore, the diffusion energy barrier of Si at O2 position was lower than that at O1 position. This complied with previous calculations of Si atom diffusion in γ′-Ni_3_Fe supercells.

#### 3.3.4. Diffusion Coefficient

According to the diffusion energy barriers and distances, the relationship of Si diffusion coefficient and temperature was determined by Arrhenius’ equation [35,36,37]. Based on the transition state theory, a functional relationship existed between the diffusion coefficient and the transition frequency, and the transition frequency met Arrhenius’ equation [38,39], which was expressed as:

(3) $$D=L^{2}\times \Gamma$$

where *D* is diffusion coefficient, *L* is diffusion distance, and Γ is transition frequency. Equation (4) states the transition frequency of impurity of atom diffusion in solids according to Wert et al. [40,41] as:

(4) $$\Gamma =\upsilon_{0}exp(-\frac{E_{a}}{k_{B}T})$$

where $\upsilon_{0}$ is the number of effective transitions of an atom per unit time. The effective transitions frequency of impurity atoms in solids is usually on the order of 10^13^ Hz [42]; therefore, $\upsilon_{0}$ was set to 10^13^ Hz in this study. When substituting Equation (4) into Equation (3) and considering $k_{B}=R/N_{A}$, atomic transition frequency and diffusion coefficient were stated as:

(5) $$D=L^{2}\upsilon_{0}exp(-\frac{{N_{A}E}_{a}}{RT})$$

where $N_{A}$ is the Avogadro constant, $E_{a}$ is the diffusion energy barrier, *T* is absolute temperature, and *R* is the constant of Ideal gas.

The Si diffusion distances in γ-Ni and γ′-Ni_3_Fe are summarized in Table 3. Substituting corresponding diffusion energy barriers into Equation (5) gave the relationships between the diffusion coefficient and temperature as:

(6) $$D_{Ni32Si(O)}=(12.7540\times 10^{-7})\exp(-\frac{166,007.7617}{RT})\;m^{2}/s$$

(7) $$D_{Ni31CrSi(O)}=(14.7756\times 10^{-7})\exp(-\frac{154,753.5170}{RT})\;m^{2}/s$$

(8) $$D_{Ni31MoSi(O)}=(11.5794\times 10^{-7})\exp(-\frac{323,757.7314}{RT})\;m^{2}/s$$

(9) $$D_{Ni31WSi(O)}=(12.2734\times 10^{-7})\exp(-\frac{396,919.0198}{RT})\;m^{2}/s$$

(10) $$D_{Ni24Fe8Si(O1)}=(12.6612\times 10^{-7})\exp(-\frac{130,067.7246}{RT})\;m^{2}/s$$

(11) $$D_{Ni24Fe7CrSi(O1)}=(12.7310\times 10^{-7})\exp(-\frac{125,722.1773}{RT})\;m^{2}/s\;$$

(12) $$D_{Ni24Fe7MoSi(O1)}=(10.5746\times 10^{-7})\exp(-\frac{287,740.7557}{RT})\;m^{2}/s$$

(13) $$D_{Ni24Fe7WSi(O1)}=(10.7509\times 10^{-7})\exp(-\frac{331,600.9144}{RT})m^{2}/s$$

(14) $$D_{Ni24Fe8Si(O2)}=(12.5454\times 10^{-7})\exp(-\frac{175,768.2371}{RT})\;m^{2}/s$$

(15) $$D_{Ni24Fe7CrSi(O2)}=(12.4822\times 10^{-7})\exp(-\frac{96,690.8376}{RT})\;m^{2}/s$$

(16) $$D_{Ni24Fe7MoSi(O2)}=(9.9690\times 10^{-7})\exp(-\frac{124527.3926}{RT})\;m^{2}/s$$

(17) $$D_{Ni24Fe7WSi(O2)}=(9.8668\times 10^{-7})\exp(-\frac{154,975.1302}{RT})\;m^{2}/s$$

**Figure 10:**
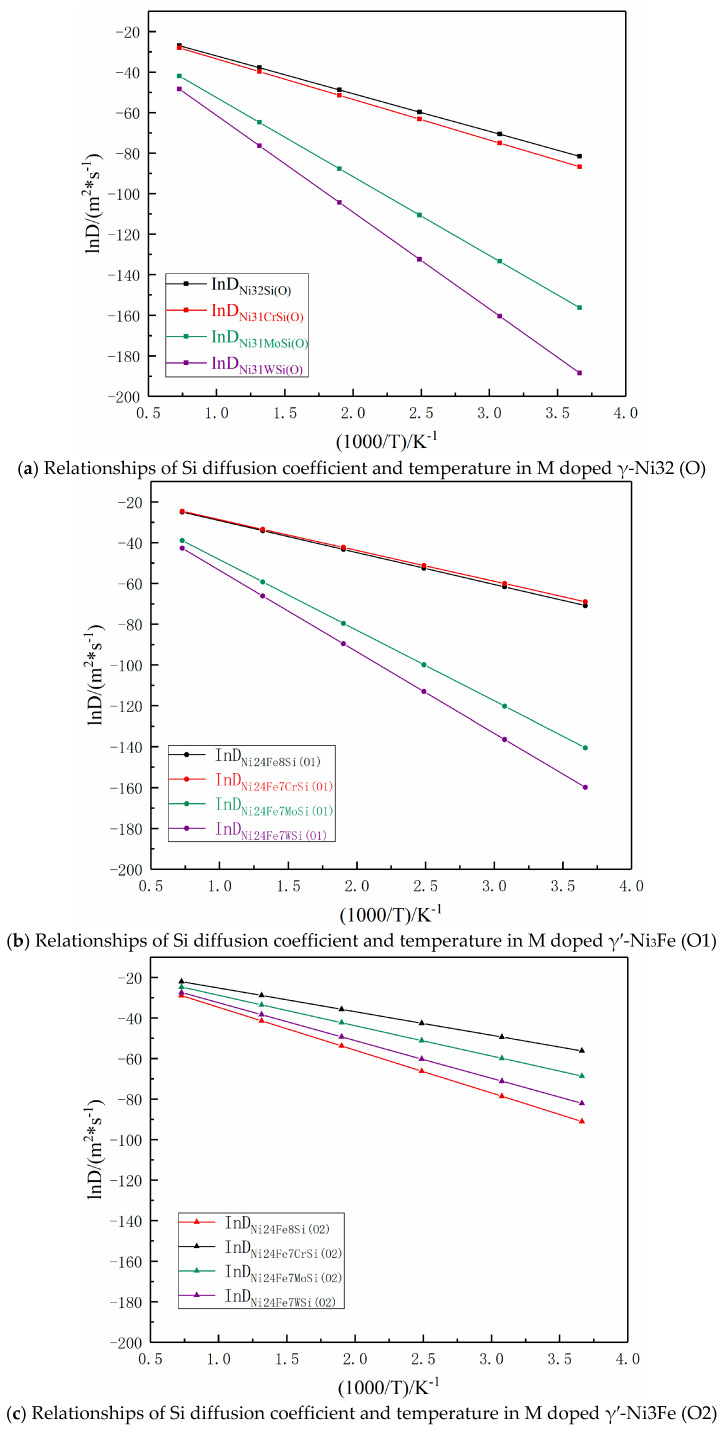
Arrhenius plots between the logarithm of the diffusion coefficient and reciprocal of temperature for Si atom diffusion in different supercells.

According to Equations (6)–(17), relationships of Si atom diffusion coefficients and temperature are shown in Figure 10 for the temperature range from 273 to 1273K. It was seen that Si atom diffusion coefficients in γ-Ni and γ′-Ni_3_Fe supercells were increased with the increase of temperature and the difference in Si atom diffusion coefficients in different supercells from low- to high-temperature were gradually decreased, especially in Ni32Si (O), Ni31CrSi (O), Ni24Fe8Si (O1), and Ni24Fe7CrSi (O1) supercell systems, where Si diffusion coefficients were very close, indicating that the doped Cr element had minimal effect on Si diffusion as the MPD element. However, Mo and W atoms hindered Si atom diffusion in Ni31MoSi(O), Ni31WSi(O), Ni24Fe7MoSi(O1), and Ni24Fe7WSi(O1) systems. It is worth noting that in the γ′-Ni_3_Fe supercell system, when O2 was the initial and final diffusion position, the doped Cr, Mo, and W played key roles in promoting Si atom diffusion. The main reason might be that O2 octahedron was composed of Fe and Ni atoms, not because γ-Ni32 (O) and γ′-Ni_3_Fe (O1) was composed of Ni atoms. The type of interstitial atoms had a greater impact on Si atom diffusion, which agreed with the findings of Section 3.3.3 regarding the density of states.

## 4. Conclusions

In this paper, the diffusion behaviors of Si atoms in γ-Ni, γ′-Ni_3_Fe were studied via first-principles and the following conclusions were drawn:

(1) Doping of alloying elements M (Cr, Mo, W) in both γ-Ni and γ′-Ni3Fe supercells significantly decreased formation enthalpy with the increase of the radius of the doped atoms and Si atoms tended to occupy more octahedral interstitial sites in γ-Ni and γ′-Ni_3_Fe;
(2) Two adjacent octahedral interstitials closest to M (Cr, Mo, W) atoms were adopted as the initial and final positions of Si diffusion in γ-Ni supercells. It was found that diffusion energy barrier was increased by increasing the atomic radius of alloying elements M, indicating that the doping of large-size atoms caused higher lattice distortions, resulting in higher diffusion energy barriers;
(3) Si atoms occupied two types of octahedral interstitial sites (O1 and O2) in γ′-Ni_3_Fe. When diffusing with O1 as the isovalent site, single-peak energy barrier distributions were observed, while Si atom diffusing with O2 as the isovalent site presented an approximately double-peak energy barrier distribution. The energy barriers of the two diffusion paths were also increased with the increase of the radius of the doped M atom, but Si diffusion energy barrier around the shared Fe atoms (O2 diffusion path) between adjacent octahedral interstices was significantly lower than that around the shared Ni atoms (O1 diffusion path). This indicated that Si atoms tended to occupy O2 octahedral interstitial sites;
(4) Increase of interaction strength between the doped M atom/Octahedron constituent atom and Si atom increased the diffusion of Si atom and decreased the diffusion energy barrier.

## Figures and Tables

**Table 1:** Total energy, formation enthalpy, lattice parameters, and volumes of γ-Ni and γ′-Ni_3_Fe with Cr, Mo, and W atom doping (the data of γ′-Ni_3_Al comes from the literature).

Unit Cell	*E*_t_ (eV)	$∆H_{f}$ (eV)	Lattice Parameters (Å)	V (Å^3^)
Ni32	−173.4105		a = b = c = 7.0116 a = b = c = 7.108 [12]	345.37 359.052 [12]
Ni31Cr	−177.9648	s	a = b = c = 7.0162	345.39
Ni31Mo	−179.6984	−0.0249	a = b = c = 7.0469	349.94
Ni31W	−181.9596	−0.0346	a = b = c = 7.0488	350.23
Ni24Fe8	−191.8737	0.0050	a = b = c = 6.9859	340.94
Ni24Al8			a = b = c = 7.183 [12]	370.735 [12]
Ni24Fe7Cr	−193.4393	0.0124	a = b = c = 6.9950	342.27
Ni24Al7Cr			a = b = c = 7.1426 [33]	
Ni24Fe7Mo	−195.0244	0.0050	a = b = c = 7.0270	346.99
Ni24Al7Mo			a = b = c = 7.1688 [33]	
Ni24Al7Mo			a = b = c = 7.146 [34]	
Ni24Fe7W	−197.2241	−0.0023	a = b = c = 7.0294	347.33
Ni24Al7W			a = b = c = 7.1702 [33]	
Ni24Al7W			a = b = c = 7.146 [34]	

**Table 2:** Formation energy, lattice parameters, and volumes of Si doping in octahedral and tetrahedral interstices.

Unit Cell	*E*_t_ (eV)	$∆E_{f}$ (eV)	Lattice Parameters (Å)	V (Å^3^)
Ni32Si(O)	−177.9082	0.8325	a = b = c = 7.0615	352.12
Ni32Si(T)	−177.0116	1.7291	a = b = c = 7.1576	366.69
Ni24Fe8Si(O1)	−195.1897	2.1774	a = b = c = 7.0747	354.1
Ni24Fe8Si(O2)	−195.7070	1.6601	a = b = c = 7.0747	354.1
Ni24Fe8Si(T)	−194.5941	2.7038	a = b = c = 7.0747	354.1

**Table 3:** Diffusion distances of Si atoms in different supercells.

Unit	Ni32Si (O)	Ni31CrSi (O)	Ni31MoSi (O)	Ni31Wsi (O)
**Distance (** **Å)**	3.57128	3.84391	3.40285	3.50334
**Unit**	**Ni24Fe8Si (O1)**	**Ni24Fe7CrSi(O1)**	**Ni24Fe7MoSi (O1)**	**Ni24Fe7Wsi (O1)**
**Distance (** **Å)**	3.55823	3.56805	3.25186	3.27886
**Unit**	**Ni24Fe8Si (O2)**	**Ni24Fe7CrSi (O2)**	**Ni24Fe7CrSi (O2)**	**Ni24Fe7W (O2)**
**Distance (** **Å)**	3.54195	3.53302	3.15737	3.14115

## Data Availability

Data will be made available on request.
